# A case of peritoneal dialysis in which SARS-CoV-2 was diagnosed by sudden hearing loss

**DOI:** 10.1080/0886022X.2021.1882493

**Published:** 2021-02-08

**Authors:** Emrah Gunay, Gunay Kozan, Enver Yuksel, Ayser Mizrakli, Ozgur Aslan, Seyhmus Kavak, Safak Kaya, Zulfukar Yilmaz

**Affiliations:** Department of Nephrology, Health Sciences University of Turkey, Diyarbakir Gazi Yasargil Training and Research Hospital, Diyarbakir, Turkey; Department of Otorhinolaryngology, Health Sciences University of Turkey, Diyarbakir Gazi Yasargil Training and Research Hospital, Diyarbakir, Turkey; Department of Microbiology, Health Sciences University of Turkey, Diyarbakir Gazi Yasargil Training and Research Hospital, Diyarbakir, Turkey; Department of Biochemistry, Health Sciences University of Turkey, Diyarbakir Gazi Yasargil Training and Research Hospital, Diyarbakir, Turkey; Department of Radiology, Health Sciences University of Turkey, Diyarbakir Gazi Yasargil Training and Research Hospital, Diyarbakir, Turkey; Department of Infectious Diseases, Health Sciences University of Turkey, Diyarbakir Gazi Yasargil Training and Research Hospital, Diyarbakir, Turkey; Department of Nephrology, Dicle University Faculty of Medicine, Diyarbakir, Turkey

Dear Editor,

Sudden hearing loss in cases of SARS-CoV-2 is rare, and it is even less common as a first symptom. For the first time in the literature, we present a case of SARS-CoV-2 accompanied by bilateral sudden hearing loss in a peritoneal dialysis patient.

A 23-year-old female patient had been on a continuous ambulatory peritoneal dialysis program for the last 5 years. Three days prior to presenting to the emergency room, she started experiencing sudden onset bilateral ear pain and hearing loss. She was admitted to the pandemic ward after infiltrative ground-glass densities showing bilateral consolidation consistent with SARS-CoV-2 pneumonia were observed in thoracic CT images. SARS-CoV-2 PCR results were positive in the nasopharyngeal and oropharyngeal swabs. She had severe lymphopenia (0.43 × 10^3^/µL), high C-reactive protein (CRP) levels (140 mg/L; normal range: 0–5 mg/L), and high D-dimer concentration (838 ng/ml). Favipiravir, enoxaparin, and meropenem were started. Immediately after the nasopharyngeal and oropharyngeal swab results were found positive, samples were taken from four consecutive dialysates with six hours of waitings. The samples were studied after being centrifuged at 4000 rpm for 10 min. The SARS-CoV-2 PCR results were negative in all peritoneal effluent samples. The main complaint that brought the patient to the hospital was bilateral ear pain and hearing loss. Bilateral serous otitis media were observed on otoscopic examination. Moderate hearing loss of mixed type (sensorineural and conductive) was observed in the audiogram. Type-B appearance in both ears was seen in tympanometry. Methylprednisolone 56 mg/day was started to be administered orally. No intratympanic steroids were given. Methylprednisolone was continued by decreasing 8 mg daily. During an examination four days later, it was observed that the patient’s complaints and the appearance of serous otitis were reduced. Mild conductive hearing loss was observed on the audiogram. She did not need oxygen during her hospitalization. Hypervolemia was not observed. The 4 × 2000 mL CAPD/DPCA 17 (Fresenius Medical Care Deutschland GmbH) treatment was continued. Hearing loss improved, and the CRP regressed to 35 mg/L. The patient, who had no additional complaints, was discharged on the seventh day of hospitalization.

The coexistence of sudden hearing loss and SARS-CoV-2 is rare, but it has been reported with increasing frequency [1–3]. Profound hearing losses that require a cochlear implant could also be seen [4]. Drugs such as hydroxychloroquine and chloroquine used in the treatment of SARS-CoV-2 are known to be ototoxic [5]. In the present case, our patient did not use these drugs. However, the complaints of hearing loss and ear pain improved with the initiation of 1 mg/kg/day oral methylprednisolone one day after hospitalization and gradually reduced steroid doses. Since sudden hearing loss is a condition that requires urgent care, due attention should be given and treatment should be started as early as possible.
